# Macular Hole Progression after Intravitreal Bevacizumab for Hemicentral Retinal Vein Occlusion

**DOI:** 10.1155/2011/679751

**Published:** 2012-01-31

**Authors:** Manish Nagpal, Vikram Mehta, Kamal Nagpal

**Affiliations:** Department of Vitreo Retina, Retina Foundation, Shahibag, Gujarat, Ahmedabad 380004, India

## Abstract

Macular edema secondary to retinal vein occlusion is commonly being treated with off-label intravitreal bevacizumab with good outcomes. A significant reduction in macular edema and improvement in visual acuity is seen following such a treatment with no serious adverse effects. In the reported case, a full-thickness macular hole was noticed one month after intravitreal bevacizumab for macular edema secondary to hemicentral retinal vein occlusion. On a detailed review of the pre- and postoptical coherence tomography scans, it was realized that there was a preexisting stage 2-3 macular hole which was masked by the hemorrhages and edema at the fovea and the macular hole had progressed following the injection.

## 1. Introduction

Intravitreal injection of bevacizumab appears to result in significant short-term improvement of visual acuity and macular edema secondary to retinal venous occlusion [1]. Bevacizumab has been reported to be well tolerated with negligible ocular toxicity or adverse effects [2].

We report our unusual experience while treating a case of hemicentral retinal vein occlusion with macular edema.

## 2. Case Report

**Figure 1:**
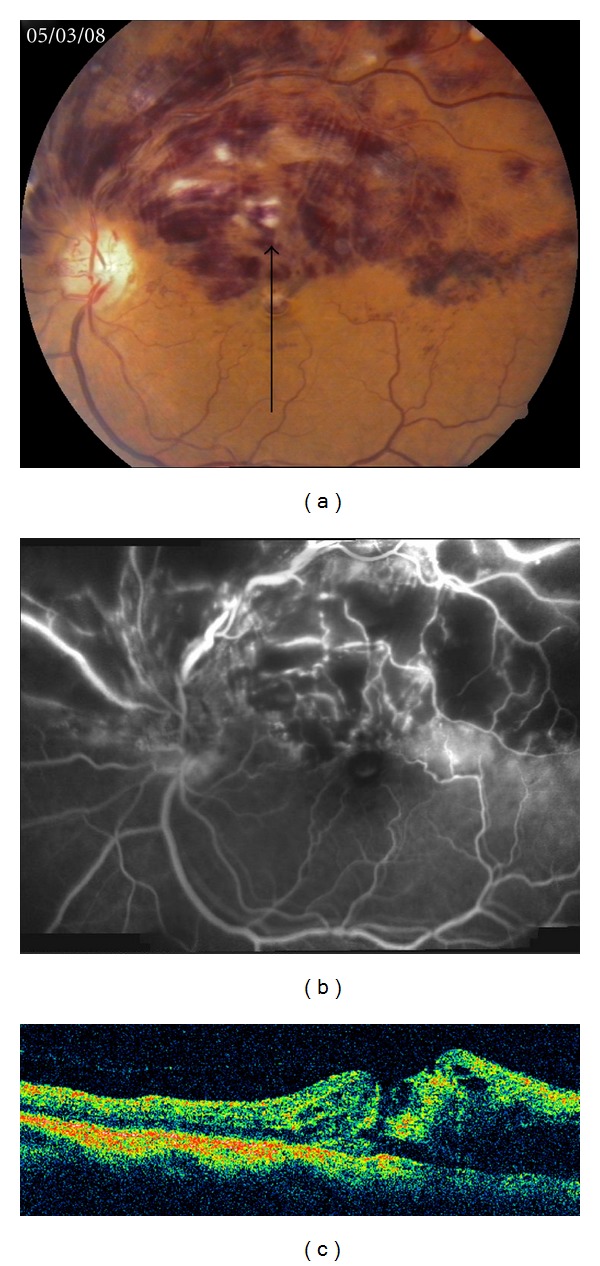
(a) Fundus photograph of the left eye showing superficial retinal hemorrhages, cotton wool spots, and tortuous and dilated retinal veins in the superior half of the retina involving the macula. These findings were consistent with a superior HCRVO. (b) Fluorescein angiography demonstrated blocked fluorescence due to retinal hemorrhages with areas of capillary non perfusion. (c) Vertical 5 mm OCT scan along the arrow shows cystoid edema with retinal hemorrhages in the superior half of the fovea (right half of the scan) and some retinal discontinuity which in retrospect was a stage 2-3 macular hole. Hyaloidal attachment is also seen.

**Figure 2:**
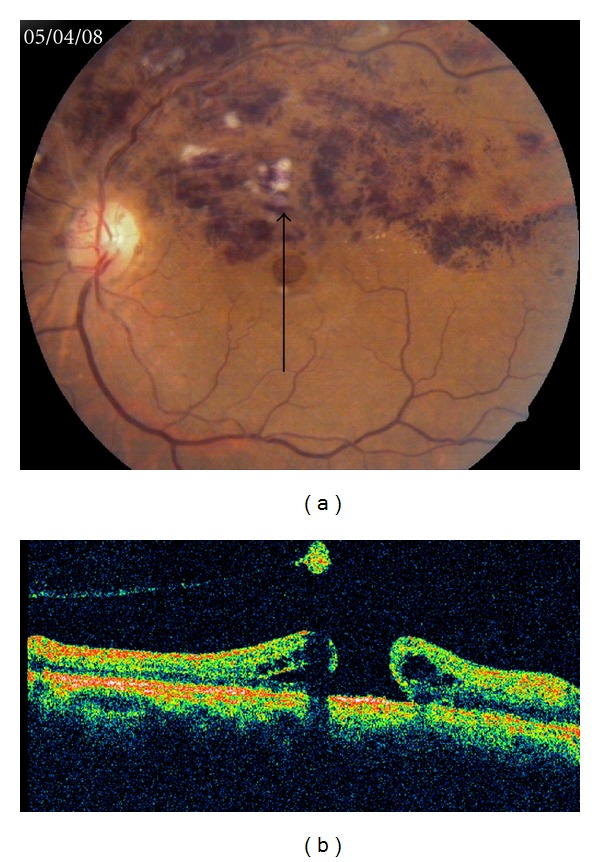
(a) Fundus photograph, one month after intravitreal bevacizumab injection, showing decreased haemorrhages with a full-thickness macular hole. (b) Vertical 5 mm OCT scan along the same line shows progression to a full-thickness macular hole with a detached hyaloid and a pseudooperculum causing a shadowing behind it.

A 60-years-old lady presented to us with complaints of decreased vision in the left eye for 2 months. Best corrected visual acuity was 20/60 in the right eye and 3/60 in the left eye with normal intraocular pressure. Anterior segment examination was unremarkable except for a grade 2 nuclear sclerosis in both eyes. Fundus examination of the left eye revealed a superior hemicentral retinal vein occlusion (HCRVO), which was documented on fluorescein angiography as well (Figures 1(a) and 1(b)). Optical coherence tomography (OCT) revealed cystoid edema with loss of normal foveal contour (Figure 1(c)). Fundus evaluation of the right eye was normal. She was treated with intravitreal injection of bevacizumab (1.25 mg) after a detailed discussion and informed consent explaining the risks involved and the off-label use. At one-month followup, there was no subjective or objective improvement in vision. Fundus examination revealed reduction in the retinal hemorrhages and a full-thickness macular hole which was evident on OCT as well (Figures 2(a) and 2(b)). Retrospectively, analyzing the preinjection OCT, it was realized that the irregular foveal margins were because of a preexisting stage 2-3 macular hole camouflaged by the hemorrhages and edema at the fovea which had progressed following the injection.

## 3. Discussion

Lamellar macular holes and rarely full-thickness macular holes are occasional complications of chronic macular edema in venous occlusions [3]. The macular hole in this case was seen in the early phase of the occlusion, and, hence, it was probably an HCRVO developing in a case of idiopathic macular hole. However, the possibility of macular edema initiating an early macular hole cannot be completely ruled out.

The intravitreal bevacizumab injection could have probably led to the progression of the macular hole. There have been isolated reports of formation/progression of macular hole following intravitreal triamcinolone for central retinal vein occlusion [4, 5]. Changes in the structure of the vitreous body induced by intravitreal injections are known. Possible peripheral vitreous pull exerted by a minimal vitreous incarceration at the injection site could account for anteroposterior traction [4]. Numerous reports have already shown that intravitreal bevacizumab induces a prompt improvement in macular edema secondary to venous occlusions as observed in our patient [1, 2]. The shrinkage of macular thickness combined with anteroposterior traction could have led to the progression of a stage 4 full-thickness macular hole.

In summary, combined HCRVO with idiopathic macular hole is a rare presentation. Diagnosis of macular hole could be difficult in such a scenario, and OCT is a useful tool for its detection. Commonly practiced treatment of macular edema for venous occlusion such as off label intravitreal bevacizumab could cause a progression of the macular hole, and, hence, careful examination of the macular OCT image should include careful scrutiny for early macular hole formation. Overall prognosis despite resolution of macular edema will be guarded due to the presence and progression of the macular hole.
